# Physicians’ Dose and Symptom Book

**Published:** 1874-03

**Authors:** 


THE PHYSICIAN'S DOSE AND SYMPTOM BOOK, *containing the doses and uses of all the principal articles of the Materia Medica and Officinal Preparations; also, table of weights and measures; rules to proportion the doses of medicines; common abbreviations used in writing prescriptions; table of poisons and antidotes; index of diseases and treatment; pharmaceutical preparations; table of symptomatology; and outlines of general pathology and therapeutics.* By Joseph H. Wythes, A.M., M.D., etc. Eleventh edition, revised. Philadelphia: Lindsay & Blakiston, 1874. 16 mo. Cloth; pp. 236—price $1.25.

All we need say of this handsome little volume is, that it is fully up with the times, very portable and very convenient to a young graduate who desires to post himself upon horseback.

